# The Recurrence of Ptosis after Correction Surgery Is Associated with Refractive Error

**DOI:** 10.3390/medicina59030630

**Published:** 2023-03-22

**Authors:** Yu Ke, Jie Meng, Min Zhou, Pengsen Wu, Fan Zhang, Xiangqing Hei, Danping Huang

**Affiliations:** 1 Zhongshan Ophthalmic Center, Sun Yat-Sen University, Guangzhou 510080, China; 2 The First Affiliated Hospital of Guangzhou Medical University, Guangzhou 510120, China

**Keywords:** ptosis, recurrence, frontalis sling surgery, refractive error

## Abstract

*Background and objectives*: Previous studies on ptosis recurrence after correction surgery have tended to focus on postoperative complications, surgical methods and suspension materials, few have mentioned refractive error. This research is to investigate the potential relation between refractive error and recurrence after correction surgery in pediatric patients with simple congenital ptosis. *Materials and Methods*: We conducted a retrospective analysis of data from patients with simple congenital ptosis who were treated at Zhongshan Ophthalmic Center (ZOC) between 2017 and 2020. In total, 111 eyelids of 85 patients without surgery-related complications who underwent frontalis muscle flap suspension (FMFS) for simple congenital ptosis were included. Postoperative changes in eyelid height were assessed. Cycloplegic refraction was assessed before surgery and during the follow-up period (every 3 months after surgery). Recurrence in the postoperative period was defined as a marginal reflex distance 1 (MRD1) of <1 mm. *Results*: There were 16 recurrence and 69 non-recurrence cases, with no statistically significant differences, in terms of patient age at the time of surgery, patient sex, or preoperative MRD1, between the recurrence and non-recurrence groups. The postoperative cylindrical diopter (adjusted odds ratio [OR] = 0.432, *p* = 0.005), laterality (adjusted OR = 0.202, *p* = 0.006), and preoperative MRD1 (adjusted OR = 0.617, *p* = 0.019) were associated with ptosis recurrence after surgery. Differences between the recurrence and non-recurrence groups in spherical diopter and spherical equivalent (SE) before and after surgery were not statistically significant. In addition, preoperative refractive error and postoperative spherical diopter were not significantly associated with ptosis recurrence after correction surgery. *Conclusions*: Ptosis recurrence after FMFS in pediatric cases of congenital ptosis is associated with refractive error. Timely refractive correction and amblyopia treatment may help to reduce ptosis recurrence.

## 1. Introduction

Congenital ptosis, defined as an abnormally low position of the upper eye lid at birth, may be simple or associated with other congenital conditions, such as Marcus Gunn syndrome, blepharophimosis, or congenital third cranial nerve palsy [1]. Congenital ptosis is known to be associated with refractive error and abnormal visual development [2,3,4,5,6,7,8]. Patients with ptosis tend to have a larger degree of astigmatism in the ptotic eye than in the non-ptotic eye [2,4,9], in addition to significant differences in corneal topography between the ptotic and non-ptotic eye [4,10,11,12,13,14]. A common indication for early surgical correction of congenital ptosis is the prevention or treatment of amblyopia, which may be due to obstruction of the visual axis and associated significant refractive error.

Many surgical procedures have been introduced to correct ptosis, including levator palpebrae superioris shortening and frontalis muscle flap suspension (FMFS), which use autologous tissue, and those that utilize frontalis suspension materials, such as silicone tubes, nylon monofilaments, banked fascia lata, and polytetrafluoroethylene [15,16]. The rate of recurrence after frontalis sling surgery ranges from 14.1% to 62.2% [16,17,18,19,20,21].

Most previous studies on ptosis recurrence have focused on its association with comorbidities, surgical complications, and patient age at the time of surgery [17,18,20,21,22,23,24,25,26]. Many studies have examined surgery-related factors, such as surgical designs and suture materials [17,18,20,22,25,27], or changes in refractive error after correction surgery [11,13,14,28,29]. There have been few studies on the relationship between refractive error and ptosis recurrence after correction surgery. Thus, the present study aimed to investigate factors other than surgical complications involved in ptosis recurrence after correction surgery, specifically, the association between refractive error and recurrence.

## 2. Methods

This study was approved by the ethics committee of Zhongshan Ophthalmic Center (ZOC) (2017KYPJ124). Informed consent was obtained at the time of patient enrollment. At the time of admission, the physician explained the plan and intent of the study to each patient (those older than 18 years) or to the patient’s guardian, and the same pre and postoperative precautions and surgical risks were explained to each patient. If the patient agreed, an informed consent form was then signed and the follow-up examination would be performed and recorded. The study adhered to the tenets of the Declaration of Helsinki.

### 2.1. Inclusion and Exclusion Criteria

The medical records of all patients diagnosed with simple congenital ptosis and treated with FMFS at ZOC between 1 January 2017 and 31 December 2019 were obtained. The inclusion criteria were as follows: (1) patients admitted for surgical treatment of congenital ptosis from 1 January 2017 and 31 December 2019 at ZOC, with no systemic disorder and no specific restrictions on age or laterality; and (2) patients themselves (older than 18 years old) or patients’ guardians gave consent for the study. The exclusion criteria were as follows: (1) aponeurotic, neurogenic, mechanical, and non-congenital forms of myogenic ptosis (e.g., muscular dystrophy, myasthenia gravis, or chronic progressive external ophthalmoplegia) and pseudoptosis; (2) patients with ocular diseases other than simple congenital ptosis that may affect visual acuity; (3) patients with a history of surgery or trauma involving the eyelids; (4) patients with complications, including punctate corneal epithelial erosion, granuloma formation, wound infection, postoperative entropion, conjunctival prolapse, poor eyelid contour, exposure to suture materials, and localized allergic reactions to suture materials.

### 2.2. Ophthalmologic Examination

Preoperative cycloplegic refraction and marginal reflex distance 1 (MRD1) were measured at the time of the first visit and again at least 3 months after surgery during the follow-up. The date of birth, date of each visit, date of surgery, and treatment methods were recorded. The refractive data were first examined using an autorefractor keratometer (KR8800; Topcon, Tokyo, Japan) under cycloplegic conditions (0.5% tropicamide and 0.5% phenylephrine hydrochloride). All the patients then underwent medical optometry, which was carried out in ZOC by an experienced optometrist. The refractive power was measured at intervals of 0.25 diopter (D). All measurements were made three times, and the average value was recorded. The diopter of sphere (DS), diopter of cylinder (DC), and spherical equivalent (SE) were measured. The SE was calculated as follows: SE = DS + ½ × DC. Cylindrical power was recorded as (−). As the majority of our patient population was aged <5 years, the results of subjective refraction and levator function measurements were difficult and unreliable due to poor cooperation. Thus, we excluded these parameters from our study. We defined MRD1, recorded in millimeters, as the distance from the margin of the upper eye lid to the central corneal light reflex. The ptosis severity was defined according to the MRD1: non-ptotic (MRD1 ≥ 3), mild-to-moderate (0 < MRD1 < 3), and severe (MRD1 ≤ 0).

### 2.3. Surgical Technique

All the surgeries were performed under general anesthesia.

**Figure 1:**
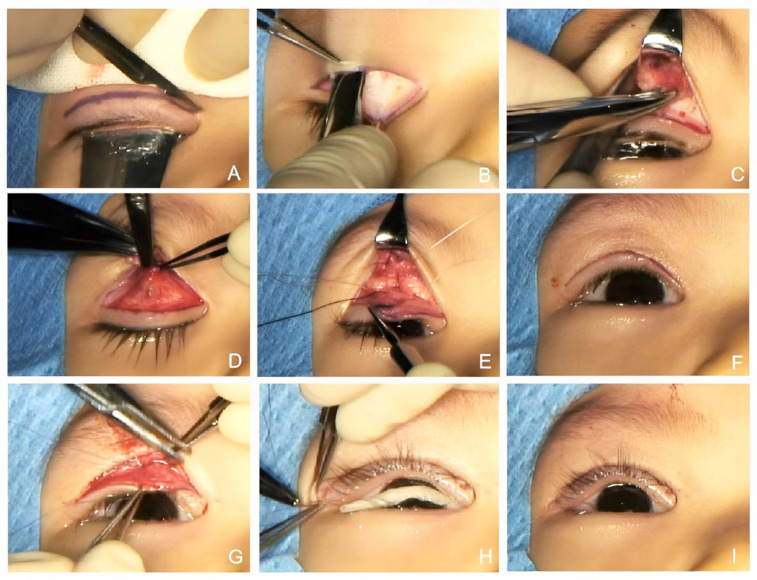
(**A**): Upper lid skin was incised along the marked crease line; (**B**): separation of eyelid skin and subcutaneous tissues; (**C**): the frontalis muscle was clamped; (**D**): a bridge-shaped strip of orbicularis oculi muscle was created; (**E**): the frontalis fascial tissue flap was fixed in the mid-superior of the tarsal plate; (**F**): the lid fissure reached a height of 8 or 9 mm and the lid margin was well curved; (**G**): the separated bridge-shaped orbicularis oculi muscle strip was fixed onto the end of the frontalis fascial tissue flap; (**H**): the skin was intermittently sutured; (**I**): the eyelid reached a satisfying height and contour at the end of surgery.

In the FMFS procedure (showed in Figure 1), an eyelid incision was placed approximately 4 mm above the lid margin and marked with methylene blue. The upper lid skin was incised along the marked crease line, and the eyelid skin and subcutaneous tissues were separated, all the way from the orbicularis oculi muscle upward to 10 mm above the brow, subcutaneously. The frontalis muscle was clamped using vascular forceps and cut vertically on both sides to create a trapezoidal frontalis muscle flap. The subcutaneous fascia tissue of the upper lid was removed from the tarsus, and part of the orbicularis oculi muscle was cut horizontally to create a bridge-shaped strip of orbicularis oculi muscle. The frontalis fascial tissue flap was transferred downward and fixed in the mid-superior of the tarsal plate to ensure that the lid fissure reached a height of 8 mm and that the lid margin was well curved. Excess muscles were then resected. The separated bridge-shaped orbicularis oculi muscle strip was fixed onto the end of the frontalis fascial tissue flap at the upper edge of the tarsal plate. The skin was intermittently sutured to produce an esthetically pleasing eyelid contour and upper eyelid crease. Care was taken to ensure that the angle of the upper lid lashes was appropriate and that the lashes did not hinder vision. The endpoint was determined intraoperatively on the basis of the central upper lid margin positioned at a desired level according to the severity of ptosis.

### 2.4. Evaluation of Recurrence

Recurrence was defined as postoperative MRD1 of <1 mm. Cases where the MRD1 dropped below 1 mm within 1 week after the surgery were defined as failed operations, and these cases were excluded. To evaluate recurrence and exclude comorbidity and postoperative complications, all the patients underwent a complete ophthalmologic examination. The same experienced ophthalmologist who had performed the initial examinations performed the postoperative follow-up examinations. The main outcome measure was postoperative recurrence of ptosis.

### 2.5. Statistical Analysis

Descriptive results are presented as mean (standard deviation [SD]) or median (interquartile range [IQR]) for continuous data and number (frequency) for categorical data. Statistical analysis was performed using a chi-square test for categorical data. The continuous variables were statistically analyzed between groups using the Wilcoxon signed-rank test or Kruskal–Wallis one way analysis of variance test, with Bonferroni correction for multiple tests. The primary comparison groups were recurrence versus non-recurrence groups. Binary logistic regression analysis was performed to evaluate the associations between recurrence (MRD < 1 mm) and other clinical parameters. The data in the single regression analysis, including study group, age, sex, and all variables significant at the level of *p* < 0.05, were included in a multiple regression model. The following covariates were studied: demographic factors, refractive error, and MRD1. A *p* value of <0.05 was considered statistically significant. All statistical analyses were performed using SPSS software version 25.0 (IBM Corp., Armonk, NY, USA)

## 3. Results

### 3.1. Demographic and Clinical Data

**Figure 2:**
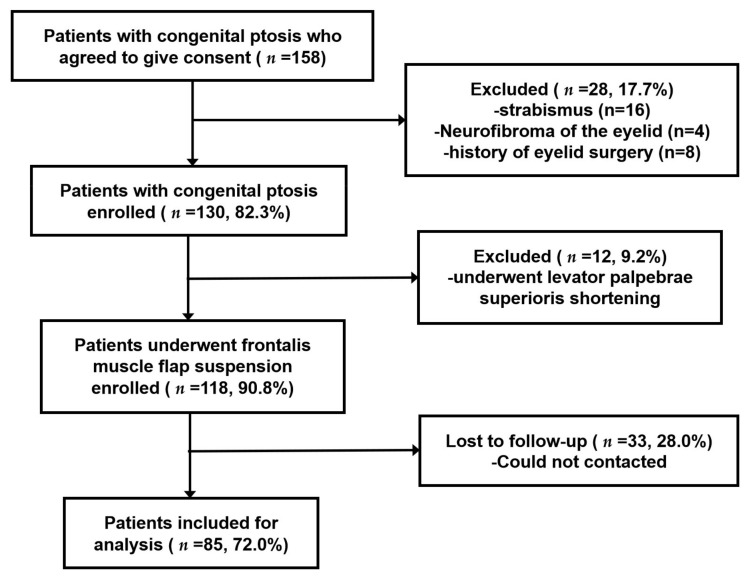
Enrollment of patients into the retrospective study on association between postoperative ptosis recurrence and refractive error.

Among 158 patients with congenital blepharoptosis, 28 (28/158; 17.7%) were excluded for having strabismus, neurofibroma of the eyelid or a history of eyelid surgery. Then, 12 patients who underwent levator palpebrae superioris shortening were also excluded. Of the 118 (118/130; 90.8%) patients enrolled in the study, 33 (28.0%) of them were lost to follow-up due to failure of contact (Figure 2). Eventually, this study included 111 eyelids of 85 patients who underwent FMFS in ZOC between May 2017 and August 2019, as displayed in Table 1, of whom 58 were males (male/female = 2.1). There were 59 (69.4%) unilateral cases and 26 (30.6%) bilateral cases in our study. The difference in sex distribution between the unilateral and bilateral groups was not significant (*p* = 0.099). In the unilateral group, the laterality of the affected eye was nearly equal (30 right eyes, 29 left eyes).

Among the unilateral cases, 30 (51%) patients were less than 5 years old, and 29 (49%) patients were more than 5 years old. In the bilateral group, 13 (50%) patients were less than 5 years old, and 13 patients were more than 5 years old. There was no significant difference in the age distribution and postoperative follow-up time between the unilateral and bilateral groups (*p* = 0.943 and *p* = 0.108, respectively).

Postoperative refraction and MRD1 were measured at a mean follow-up of 293.63 days. There were 8 (13.6%) mild-to-moderate cases and 51 (86.4%) severe cases in the unilateral group and 3 (11.5%) mild-to-moderate cases and 23 (88.5%) severe cases in the bilateral group. (Severity grouping was based on the severity of the eye with the smaller MRD1). The preoperative MRD1 in the unilateral group was significantly less than that in the bilateral group (*p* = 0.014), and the preoperative and postoperative DS in the unilateral group were significantly larger than those in the bilateral group (*p* = 0.039 and *p* = 0.013, respectively). The preoperative and postoperative DC were not significantly different in the unilateral and bilateral groups (*p* = 0.438, *p* = 0.915, respectively). The difference in the postoperative MRD1 of the two groups was not statistically significant (*p* = 0.087). In both groups, there was a significant difference in the postoperative MRD1 versus the preoperative MRD1 (*p* < 0.001).

### 3.2. Comparison of Cylindrical Power between Different Severity Groups

**Figure 3:**
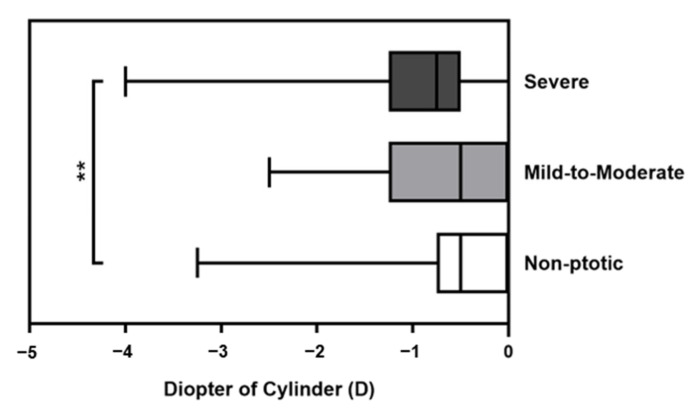
Box graph illustrating the distribution of preoperative cylinder diopter in the groups according to ptosis severity. Cylindrical power was significantly different between non-ptotic and severe groups (** *p* < 0.01, adjusted using Bonferroni correction for multiple testing).

In the comparison of cylinder power, the severity grading was classified as non-ptotic, mild-to-moderate, or severe according to preoperative MRD1. There were three unilateral and eight bilateral cases in the mild-to-moderate group and 23 unilateral and 51 bilateral cases in the severe group (Table 2). Age at surgery, sex distribution, and follow-up time were not significantly different between the mild-to-moderate group and the severe group. With regard to refractive error, the preoperative DS in the severe group was significantly larger than in the mild-to-moderate group (*p* = 0.038). The median DC values in the non-ptotic, mild-to-moderate, and severe groups were −0.50 D, −0.50 D, and −0.75 D, respectively (Figure 3). The adjusted analysis revealed significant differences in astigmatism between the non-ptotic and severe groups (*p* = 0.004). However, there was no significant difference in astigmatism in the mild-to-moderate group versus the non-ptotic group (*p* = 0.578) or severe group (*p* = 0.450).

### 3.3. Comparison of Clinical Data between the Recurrence and Non-Recurrence Groups

**Figure 4:**
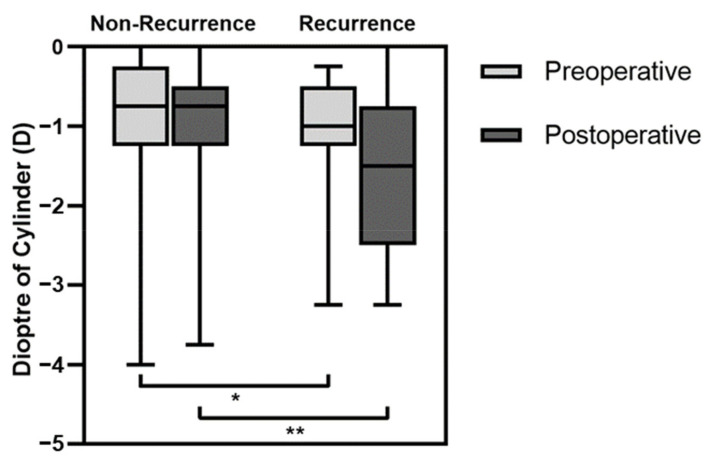
Box graph illustrating the preoperative and postoperative cylindrical power in the non-recurrence and recurrence groups. * *p* < 0.05, ** *p* < 0.01.

The differences in age, sex, follow-up time, and MRD1 were not statistically different in the recurrence and non-recurrence groups (Table 3). The proportion of bilateral cases was higher in the recurrence group than in the non-recurrence group, and the difference was statistically significant (*p* = 0.032). When the optometry data were compared, the preoperative cylindrical power in the recurrence group was significantly greater than in the non-recurrence group (−1.00 D [−1.25 D to −0.50 D] vs. −0.75 D [−1.25 D to −0.25 D], *p* = 0.042, as shown in Figure 4). The median preoperative spherical diopter was 2.25 in the recurrence group and 1.75 in the non-recurrence group, and the SE was 1.63 and 1.50, respectively, in the recurrence and non-recurrence groups, but the differences were not statistically significant (*p* = 0.115 and *p* = 0.299, respectively). Similarly, the postoperative cylindrical power in the recurrence group was significantly larger compared with the non-recurrence group (−1.50 [−2.50 to −0.75] vs. −0.75 [−1.25 to −0.50], *p* = 0.009). However, there were no statistically significant differences in postoperative spherical diopter or SE (*p* = 0.329 and *p* = 0.757, respectively).

### 3.4. Association between Recurrence and Clinical Data

The univariate analysis showed that laterality, preoperative MRD1, and postoperative DC were significantly correlated with ptosis recurrence (Table 4). The multivariate analysis showed that laterality (adjusted OR = 0.202), preoperative MRD1 (adjusted OR = 0.019), and postoperative DC (adjusted OR = 0.005) were significantly correlated with the recurrence of ptosis. There was no significant difference in the distribution of sex, age at surgery, or preoperative and postoperative DS and SE with regard to recurrence.

## 4. Discussion

This study shows, for the first time, that refractive error is associated with ptosis recurrence after FMFS. Previous studies evaluated the roles of different surgical designs and suture materials in ptosis recurrence. Wagner et al. [22] reported ptosis recurrence in 40.5% of cases after the use of polyfilament cable-type sutures and in 8.3% of cases after the use of fascia lata. They attributed ptosis recurrence in the patients treated with the polyfilament cable-type sutures to inadequate suture–tissue bonds and granuloma formation. Ptosis recurrence can also be due to degradation of sutures by hydrolysis after implantation [25]. In terms of the materials used in ptosis surgery, Simon et al. [17] showed that the change in MRD1 after surgery with a single loop and double pentagon sling was similar and that the lowest percentage of ptosis recurrence (15%) was achieved using polytetrafluoroethylene. All these studies focused on the association between ptosis recurrence and complications, suture materials, or surgical methods. However, in the absence of surgical complications, recurrence of ptosis after correction surgery occurs in a proportion of patients. Therefore, we speculate that, in addition to these previously investigated complications, other factors may account for ptosis recurrence after correction surgery. Manners et al. [23] suggested that recurrent ptosis with Prolene sutures was due to the sutures “cheese wiring” through the tissues. Hayashi et al. [20] reported slippage of nylon sutures from the tarsus in 92.9% of recurrence cases during reoperations, proving support for this suggestion. In their study, after reopening the surgical sites in patients with postoperative recurrence, the suspension sutures were pulled away from the original mid-superior site of the tarsus to the superior margin of the tarsus. It is feasible to suppose that this change in the suspension material clearly has an important effect on postoperative recurrence of ptosis.

Excessive forceful eye closure should be avoided after ptosis correction surgery, especially in the early postoperative period. Over blinking or eye-closing movements can place excessive pulling force on the fixation material, which can affect the position of the sutures or sling material, subsequently leading to a change in the postoperative MRD1 and ultimately ptosis recurrence. In our study, we found no significant difference in the preoperative MRD1 between the recurrence and non-recurrence groups, although preoperative and postoperative astigmatism were significantly larger in the recurrence group. The multivariate analysis also showed a significant association between postoperative DC and ptosis recurrence. Based on the findings of our study, it is reasonable to speculate that, in patients with a large degree of astigmatism, squinting the eye to obtain clearer vision may lead to a decrease in postoperative MRD1. In our study, we did not include best-corrected visual acuity data, as most of the participants in our study were too young to cooperate. However, according to previous research, the risk of amblyopia development is increased in ptotic eyes [3,5,8,9,15,30]. Amblyopia may develop in an affected eye with a large degree of astigmatism, and the patient tends not to use the amblyopic eye as a result. A previous study found that ptosis recurrence in amblyopic eyes may be due to a lack of visual drive on the affected side [31]. All these refractive error-induced movements of the orbital muscle probably lead to ptosis recurrence.

In our study, the differences in preoperative SE between the different severity groups were not statistically significant, which is similar to the findings of some [29,32], but not all, studies [3,4,5,7]. Hoyt et al. [7] reported monocular axial myopia of the affected eye in eight infants (age range: 7 weeks to 19 months) with neonatal eyelid closure. In a cross-sectional study (*n* = 33,103, age distribution from <20 years to >60 years), Kim et al. [4] reported hyperopic shift in ptotic eyes compared to non-ptotic eyes. An increased risk of developing myopia, as well as hyperopia, has been found in children with ptosis [5]. In a study on 41 patients (age range 3–15 years), Zeng et al. [3] found that the ratios of axial length to corneal curvature radii of ptotic eyes were significantly smaller than those of fellow eyes in children with unilateral ptosis. In light of these findings, they suggested that ptosis may lead to delayed eyeball development and hyperopic refractive power. In a study on a small sample (*n* = 37) of patients with unilateral ptosis (age range: 7 months to 58 years), the authors found no significant differences in axial length between the ptotic eyes and fellow eyes [32]. The discord in the findings is likely due to different sample sizes and age distribution.

The effect of patient age on refractive status cannot be neglected. Refractive status is strongly associated with age [33], especially in childhood when visual development is rapid. Different age compositions of cohorts can affect the results of studies. We conjecture that the effect of ptosis on the overall refractive state of the eye varies with time and severity. As such, the age of the study population must be considered when drawing inferences about factors affecting refractive status. The impact of this variable on ptosis recurrence requires further study.

In our study, the DC of eyes with severe ptosis was significantly larger than that of eyes without ptosis, which is in accordance with the findings of previous studies [3,4,5,6,8]. Kim et al. [4] found that a decrease in MRD1 and an increase in body mass index were associated with higher astigmatism. In our study, we found no difference in the astigmatic power in the mild-to-moderate ptosis group versus that in the group without ptosis or the severe ptosis group. Several factors, such as MRD1 and eyelid weight, can affect cylindrical power. When the upper eyelid is low and heavy, it is possible that the resulting mechanical pressure causes a significant change in corneal refractive power.

Interestingly, our analysis showed that patients with bilateral ptosis are more likely to experience postoperative ptosis recurrence than patients with unilateral ptosis. Similar results were reported in a previous study [18]. Previous research reported a decreased amount of striated muscle and fiber damage indicators in aponeuroses obtained from patients with simple congenital ptosis [34]. Heisel et al. [31] reported histological disorganization and fibrosis in the orbital septum of patients with congenital ptosis and poor levator function. It is reasonable to speculate that histological lesions in the orbital diaphragm may be more extensive and more severe in bilateral than unilateral cases. These fibrotic orbital lesions may diminish the effect of suspension of the frontalis muscle, leading to differences in postoperative recurrence rates between unilateral and bilateral affected patients. More comprehensive data and in-depth studies are needed to shed light on this issue.

Our study has some limitations due to its retrospective design. Based on our study, we can only speculate that delayed correction of refractive error may be a cause of postoperative ptosis recurrence. A longer follow-up study is needed to clarify this issue. In addition, a prospective study is needed to explore the causal relationship between refractive error and ptosis recurrence. The preoperative MRD1 in the unilateral group in our study was smaller than in the bilateral group, which could have caused some bias. However, postoperative recurrence was still higher in the bilateral group than in the unilateral group, which supports the idea that bilateral ptosis is a risk factor for recurrence. Patient loss to follow-up in our study may also have introduced some bias. In addition, the exclusion of levator function and best-corrected visual acuity from this study, due to the young age and therefore poor cooperation of our patients, has implications for our findings.

## 5. Conclusions

In conclusion, our study suggests that postoperative astigmatism is significantly associated with ptosis recurrence after FMFS in patients with simple congenital ptosis. Timely refractive correction or amblyopia treatment may help decrease the recurrence rate of ptosis after correction surgery. We recommend that patients should receive refractive correction or adhere to amblyopia treatment, such as masking, as soon as possible after the completion of surgery, especially in children under 12 years old.

## Figures and Tables

**Table 1:** Demographic characteristics of the patients.

	Unilateral (*n* = 59, 69.4%)	Bilateral (*n* = 26, 30.6%)	*p*
Sex, *n* (%)			0.099
Male	37 (62.7%)	21 (80.8%)	
Female	22 (37.3%)	5 (19.2%)	
Age, *n* (%)			0.943
<5 years	30 (50.8%)	13 (50.0%)	
≥5 years	29 (49.2%)	13 (50.0%)	
Age, mean ± SD ^a^			
Total: 6.11 ± 3.99	5.37 ± 3.12	6.96 ± 4.72	0.198
Follow-up time (days, IQR)	287 (155 to 384)	208.5 (101.5 to 376.75)	0.108
Severity, *n* (%)			0.798
Mild-to-moderate	8 (13.6%)	3 (11.5%)	
Severe	51 (86.4%)	23 (88.5%)	
Preoperative MRD1 (IQR) ^a^	−1 (−1 to 0)	0 (−1 to 0.88)	0.014
Preoperative DS (IQR) ^a^	2.00 (1.50 to 3.00)	1.75 (0.56 to 2.44)	0.039
Preoperative DC (IQR) ^a^	−0.75 (−1.50 to −0.50)	−0.75 (−1.25 to −0.25)	0.438
Preoperative SE (IQR) ^a^	1.63 (0.75 to 2.38)	1.31 (0.28 to 2.00)	0.068
Postoperative MRD1 (IQR) ^a^	2 (1.5 to 3)	2 (0 to 2.88)	0.087
Postoperative DS (IQR) ^a^	2.25 (1.50 to 3.00)	1.50 (0.50 to 2.25)	0.013
Postoperative DC (IQR) ^a^	−0.75 (−1.75 to −0.50)	−1.00 (−1.50 to −0.50)	0.915
Postoperative SE (IQR) ^a^	1.63 (0.88 to 2.50)	1.00 (−0.25 to 2.09)	0.014

Male:female ratio = 2:1. Ptosis severity grading: non-ptotic = MRD1 ≥ 3 mm; mild-to-moderate = 0 mm < MRD1 < 3 mm; severe = MRD1 ≤ 0 mm. DS = diopter of sphere; DC = diopter of cylinder; SE = spherical equivalent = DS + ½ × DC; the unit of DS, DC and SE is D. MRD1 = marginal reflex distance 1; ^a^ Wilcoxon’s signed-rank test.

**Table 2:** Comparison of variables between different ptosis severity groups.

	Mild-to-Moderate Group (*n* = 11, 12.9%)	Severe Group (*n* = 74, 87.1%)	*p*
Age (IQR) ^a^	6.52 (4.63 to 7.36)	4.70 (3.62 to 6.44)	0.050
<5 years/≥5 years ^b^	3/8	40/34	0.097
Male/female ^b^	6/5	52/22	0.296
Follow-up time (IQR) ^a^	246 (155 to 386)	243.5 (139.75 to 379.75)	0.455
Unilateral/bilateral ^b^	3/8	23/51	0.798
Preoperative DS (IQR) ^a^	1.50 (0.50 to 2.06)	2.00 (1.13 to 2.86)	0.038
Preoperative DC (IQR) ^a^	−0.63 (−1.25 0.00)	−0.75 (−1.25 to −0.50)	0.303
Preoperative SE (IQR) ^a^	1.25 (0.00 to 1.66)	1.50 (0.56 to 2.31)	0.051
Postoperative MRD1 (IQR) ^a^	2.00 (1.75 to 3.00)	2.00 (1.00 to 3.00)	0.113
Postoperative DS (IQR) ^a^	1.50 (0.25 to 2.25)	2.00 (1.00 to 2.86)	0.144
Postoperative DC (IQR) ^a^	−0.75 (−1.44 to −0.50)	−1.00 (−1.63 to −0.50)	0.832
Postoperative SE (IQR) ^a^	1.06 (−0.34 to 1.94)	1.50 (0.50 to 2.25)	0.102

IQR = interquartile range. MRD1 = marginal reflex distance 1. DS = diopter of sphere; DC = diopter of cylinder; SE = spherical equivalent = DS + ½ × DC. The unit of DS, DC and SE is D. ^a^ Wilcoxon’s signed-rank test; ^b^ Chi-square test.

**Table 3:** Comparison of different variables in the recurrence and non-recurrence groups.

	Recurrence Group (*n* = 16, 19.8%)	Non-Recurrence Group (*n* = 69, 81.2%)	*p*
Age (IQR) ^a^	4.86 (3.52 to 7.62)	4.96 (3.81 to 6.62)	0.982
<5 years/≥5 years ^b^	8/8	35/34	0.985
Male/female ^b^	11/5	47/22	0.961
Follow-up time (IQR) ^a^	217 (103 to 379)	240 (143 to 381)	0.867
Unilateral/bilateral ^b^	7/9	52/17	0.032
Preoperative MRD1 (IQR) ^a^	−1 (−2 to 0)	0 (−1 to 0)	0.067
Preoperative DS (IQR) ^a^	2.25 (1.25 to 3.50)	1.75 (0.81 to 2.50)	0.115
Preoperative DC (IQR) ^a^	−1.00 (−1.25 to −0.50)	−0.75 (−1.25 to −0.25)	0.042
Preoperative SE (IQR) ^a^	1.63 (0.50 to 2.50)	1.50 (0.50 to 2.09)	0.299
Postoperative MRD1 (IQR) ^a^	0 (0 to 0)	2 (2 to 3)	<0.001
Postoperative DS (IQR) ^a^	2.25 (0.75 to 3.75)	1.75 (1.00 to 250)	0.329
Postoperative DC (IQR) ^a^	−1.50 (−2.50 to −0.75)	−0.75 (−1.25 to −0.50)	0.009
Postoperative SE (IQR) ^a^	1.50 (−0.13 to 3.00)	1.43(0.50 to 2.13)	0.757

IQR = interquartile range. MRD1 = marginal reflex distance 1. DS = diopter of sphere; DC = diopter of cylinder; SE = spherical equivalent = DS + ½ × DC. The unit of DS, DC and SE is D. ^a^ Wilcoxon’s signed-rank test; ^b^ Chi-square test.

**Table 4:** Logistic regression analysis of factors related to ptosis recurrence.

	Single Regression			Multiple Regression	
	OR (95% CI)	*p*		Adjusted OR (95% CI)	*p*
Sex ^a^ (male as reference)	0.842 (0.298 to 2.375)	0.745			0.664
Age ^a^	1.040 (0.941 to 1.150)	0.441			0.972
Preoperative SE (IQR) ^a^	1.180 (0.898 to 1.553)	0.235			
Preoperative DS (IQR) ^a^	1.235 (0.943 to 1.617)	0.125			
Preoperative DC (IQR) ^a^	0.662 (0.399 to 1.098)	0.110			
Laterality ^a^ (bilateral as reference)	0.303 (0.113 to 0.811)	0.017		0.202 (0.064 to 0.638)	0.006
Preoperative MRD1 (IQR) ^a^	0.723 (0.508 to 1.029)	0.072		0.617 (0.412 to 0.924)	0.019
Follow-up time ^a^	1.000 (0.998 to 1.002)	0.857			
Postoperative SE (IQR) ^a^	1.099 (0.861 to 1.404)	0.448			
Postoperative DS (IQR) ^a^	1.171 (0.921 to 1.489)	0.198			
Postoperative DC (IQR) ^a^	0.512 (0.311 to 0.842)	0.008		0.432 (0.241 to 0.776)	0.005

IQR = interquartile range. MRD1 = marginal reflex distance 1. DS = diopter of sphere; DC = diopter of cylinder; SE = spherical equivalent = DS + ½ × DC. OR = odds ratio; CI = confidence interval. ^a^ Binary logistic regression.

## Data Availability

The data presented in this study are available on request from the corresponding author. The data are not publicly available due to privacy and ethical reasons.
